# Neoadjuvant radiotherapy combined with targeted and immune therapies achieves a pathological complete response in borderline resectable gallbladder cancer: a case report and literature review

**DOI:** 10.3389/fonc.2025.1574329

**Published:** 2025-05-23

**Authors:** Tao Li, Zhiqin Li, Keren Li, Gong Li, Guangxin Li, Ying Zhao

**Affiliations:** Department of Radiation Oncology, Beijing Tsinghua Changgeng Hospital, Tsinghua University, Beijing, China

**Keywords:** gallbladder cancer, radiotherapy, targeted therapy, ICIS, case report

## Abstract

**Background:**

Gallbladder cancer, a malignant tumor with a notable prevalence, is primarily treated with surgical R0 resection, which remains the most efficacious therapeutic strategy. Achieving this level of resection is particularly challenging for patients diagnosed at intermediate or advanced stages. Numerous clinical studies focusing on preoperative translational therapies, predominantly those utilizing chemotherapy, have substantiated their capacity to increase surgical resection and survival rates of patients with gallbladder cancer, despite the persistently low rate of R0 resection. The emergence of targeted therapies and immune checkpoint inhibitors (ICIs) in the postchemotherapy era, in conjunction with localized radiotherapy, has led to promising outcomes in preoperative treatment studies across a spectrum of solid tumors.

**Case:**

This article describes a case of gallbladder cancer that was deemed critically resectable and confirmed pathologically through fine-needle aspiration biopsy. The patient underwent a novel regimen of preoperative radiotherapy complemented by targeted therapy with lenvatinib and immune checkpoint inhibitors (ICIs), specifically a PD-1 inhibitor. Eight weeks postradiotherapy, a radical surgical procedure was conducted, culminating in R0 resection and the attainment of complete pathological remission.

**Conclusion:**

This case underscores the potential of integrating radiotherapy with targeted therapies and ICIs as a translational treatment approach capable of facilitating successful R0 resection in patients with critically resectable gallbladder cancer, with the added benefit of achieving complete pathological remission.

## Introduction

Gallbladder cancer represents the most common malignancy of the biliary tract, constituting approximately two-thirds of all biliary tract cancers. According to the World Health Organization, there are an estimated 120,000 new cases globally each year (1). The pathological characteristics of gallbladder cancer are highly variable. Owing to its unique anatomical location and the insidious, nonspecific nature of its symptoms, the majority of patients are typically in an advanced stage at initial presentation, leading to a poor prognosis. The 5-year overall survival rate is distressingly low, at only approximately 5% (2).

Both domestic and international experts concur that surgery is the central modality for treating gallbladder cancer. Radical resection is the sole means for patients to achieve long-term survival, with significantly prolonged survival for those who undergo R0 resection (3). The National Comprehensive Cancer Network (NCCN) guidelines recommend considering preoperative conversion therapy for gallbladder cancer patients with stage T3/T4 disease or lymph node metastasis. Numerous clinical studies have confirmed that preoperative conversion therapy can effectively increase surgical resection and survival rates in patients with gallbladder cancer (4, 5). However, the traditional neoadjuvant regimen, which is primarily based on gemcitabine chemotherapy, has only enabled one-third of patients to achieve R0 resection through conversion therapy. Therefore, there is an ongoing need to identify superior conversion therapy strategies to increase the chances of R0 surgical resection.

In this case report, we present the successful application of a combined treatment approach integrating radiotherapy, lenvatinib, and immune checkpoint blockade as a translational therapy for critically resectable gallbladder cancer. This triple therapy not only achieved R0 resection but also resulted in complete pathological remission, demonstrating significant efficacy and contributing to a promising outcome.

## Case presentation

### Chief complaints

The patient, a 65-year-old female, was diagnosed with gallbladder cancer two weeks prior to our report.

### History of present illness

**Figure 1:**
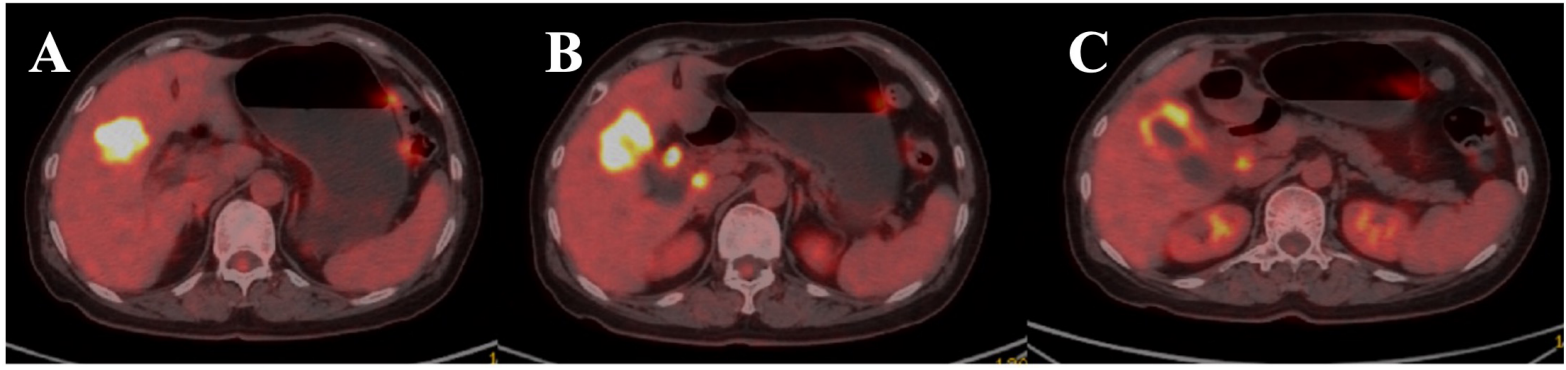
Positron emission tomography-computed tomography (PET-CT) axial image of the upper abdomen. **(A)** Hypermetabolic lesions in the gallbladder (GB), intrahepatic regions. **(B, C)** hepatoportal lymph nodes (HP LNs) are shown in the image.

In June 2023, the patient presented with right upper quadrant abdominal pain. A percutaneous biopsy revealed moderately differentiated adenocarcinoma of the gallbladder. A PET–CT scan revealed hypermetabolic lesions in the gallbladder, intrahepatic, and hepatoportal regions (**Figure 1**). The levels of the tumor markers CEA and CA19–9 were elevated.

### Personal and family histories

No significant findings were reported.

### Treatment and efficacy

Following a multidisciplinary consultation at our institution and in light of the patient’s diagnostic workup, the case was deemed borderline resectable. Preoperative conversion therapy was recommended. However, the patient and her family declined chemotherapy, and, on the basis of previous clinical practice, opted for a novel regimen of preoperative radiotherapy combined with lenvatinib and PD-L1 inhibitor immunotherapy.

The patient underwent four-dimensional computed tomography (4D-CT) simulation for treatment planning. The respiratory cycle was segmented into 0%-90% phases on the basis of respiratory signals and reconstructed via the Elekat system from Sweden. A thermoplastic shell was utilized for custom immobilization during simulation. Radiotherapy (RT) was administered via the volumetric modulated arc therapy (VMAT) technique with daily guidance from cone-beam CT images aligned with the target volume.

**Figure 2:**
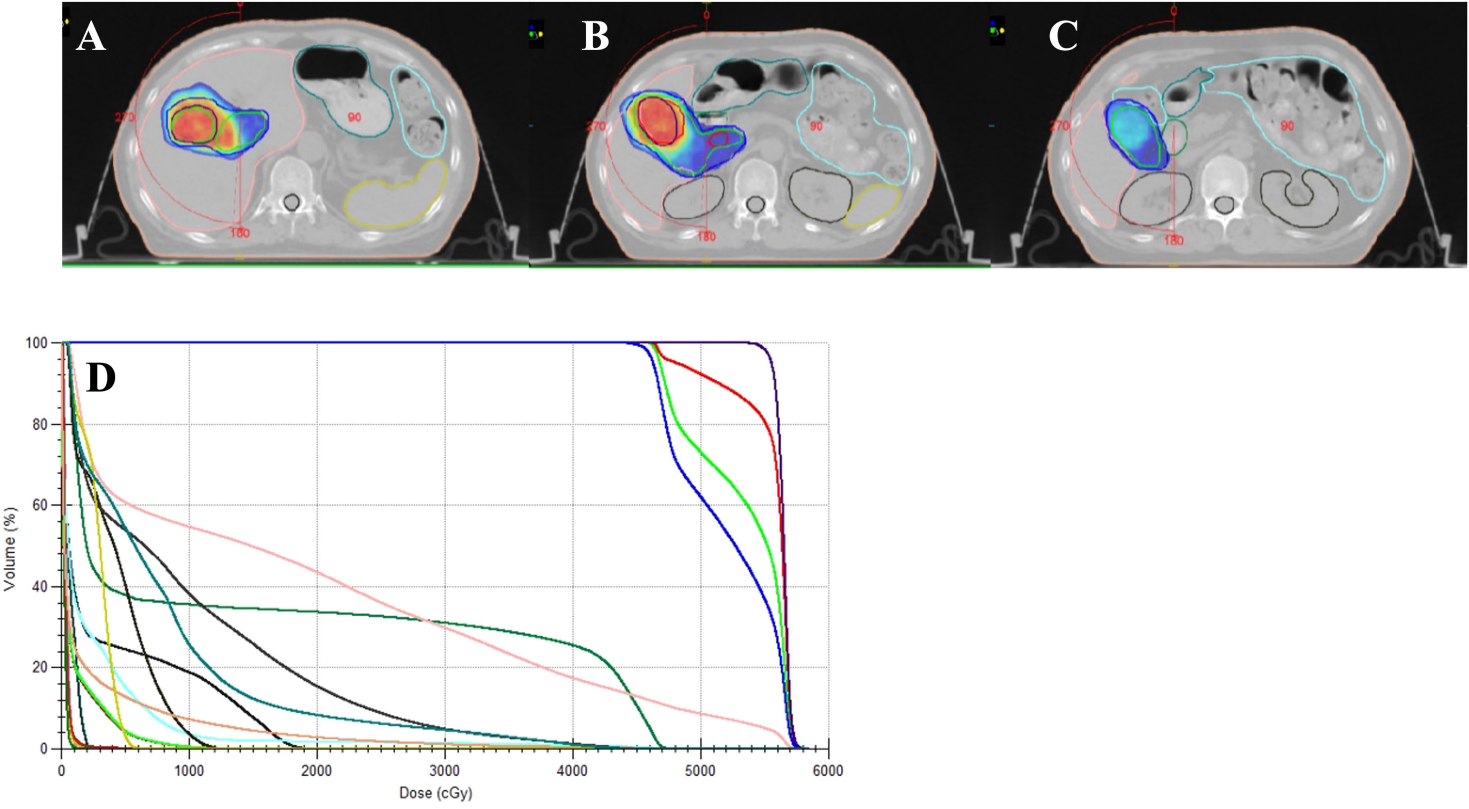
The plan of radiotherapy of the patient. **(A–C)**. Gross tumor volume (GTV) and internal target volume (ITV). Axial CT image demonstrating the delineation of the gross tumor volume (GTV) of the gallbladder lesion (orange contour) and metastatic lymph nodes (blue contour). The internal target volume (ITV) is shown as the green contour, encompassing all GTVs from the ten respiratory phases and reflecting tumor motion due to respiration. Planning target volume (PTV). Axial view showing the planning target volume (PTV) outlined in red, created by expanding the ITV by 5 mm in all directions to account for setup uncertainties and organ motion. **(D)** Dose–volume histogram (DVH) for the radiotherapy treatment plan of the patient discussed in the case report.

The gross tumor volume (GTV) of the gallbladder lesions was delineated as visible tumors and metastatic lymph nodes on the fused CT and PET–CT images. The internal target volume (ITV) was the fusion of all GTVs from the ten respiratory phases. The planning target volume (PTV) was created by expanding the ITV by 5 mm in all directions. The clinical tumor volume (CTV) included the ITV and the corresponding lymphatic drainage area. The primary tumor’s PTV was formed by expanding the CTV by 5 mm in all directions (**Figure 2A**). At least 95% of the PTV was covered by the prescribed dose defined at the periphery of the PTV (**Figure 2B**).

**Figure 3:**
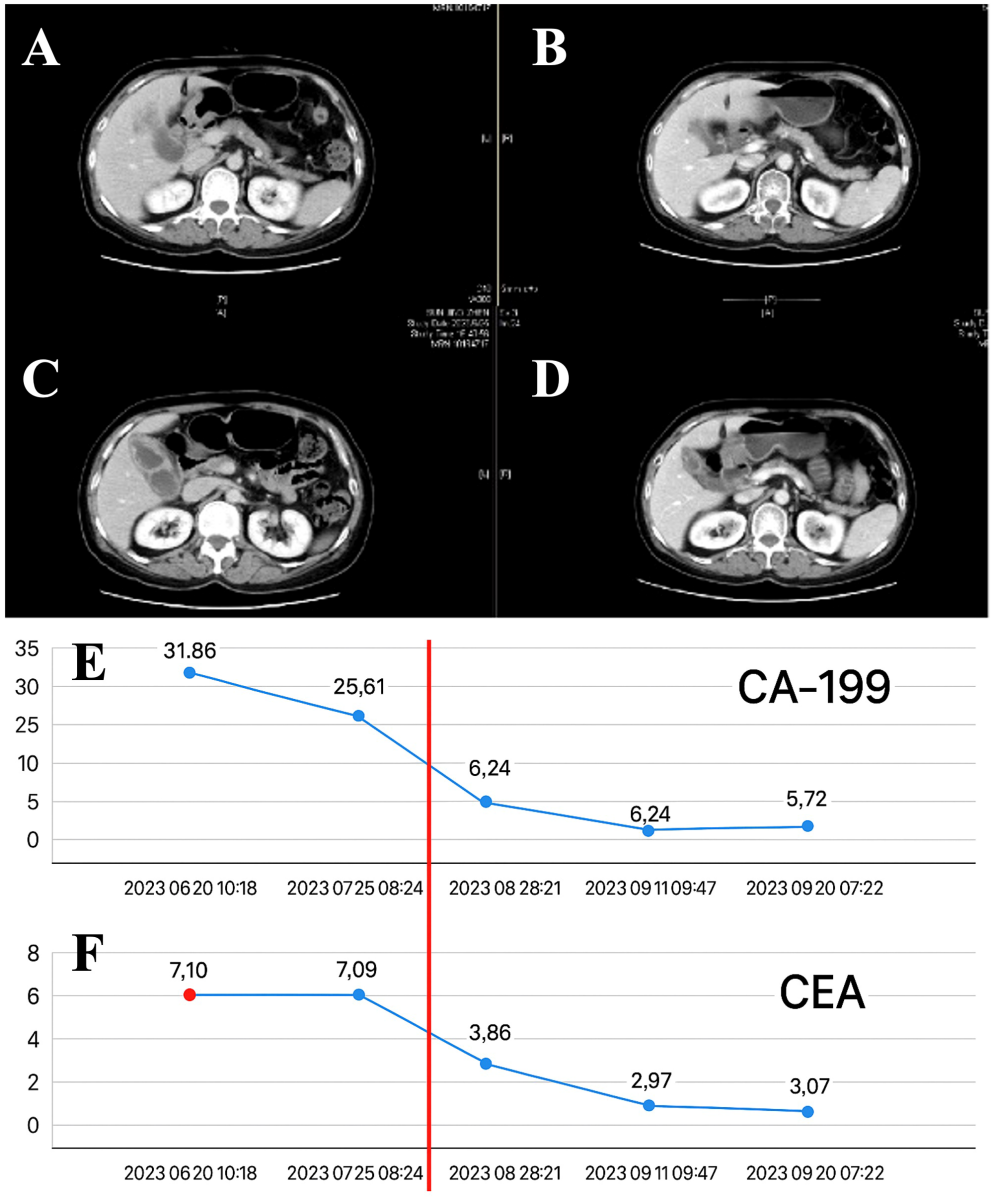
The effect after radiotherapy. **(A–D)**. The radiological images obtained before and after the neoadjuvant treatment, which consisted of radiotherapy combined with targeted and immune therapies, were compared. **(E)** The levels of carbohydrate antigen 19-9 (CA19-9). **(F)** The levels of tumor markers, specifically carcinoembryonic antigen (CEA) over time during the neoadjuvant treatment phase for critically resectable gallbladder cancer.

Radiotherapy was administered from July 10, 2023, to August 10, 2023, with the following doses: PGTV received 300 cGy × 20 fractions; PTV received 2300 cGy × 20 fractions. Lenvatinib (8 mg once daily) and sindilizumab (200 mg IV every three weeks) were administered orally at the start of radiotherapy and continued consecutively for two months after RT. In September 2023, two months after radiotherapy, an enhanced CT scan of the abdomen revealed a significant reduction in the size of the gallbladder tumor and metastatic lymph nodes compared with the previous state (**Figure 3A**). The levels of the tumor markers CEA and CA19–9 significantly decreased to within the normal range (**Figure 3B**). No grade III or higher side effects were observed during treatment. According to the Response Evaluation Criteria in Solid Tumors (RECIST version 1.1), the patient achieved partial remission.

**Figure 4:**
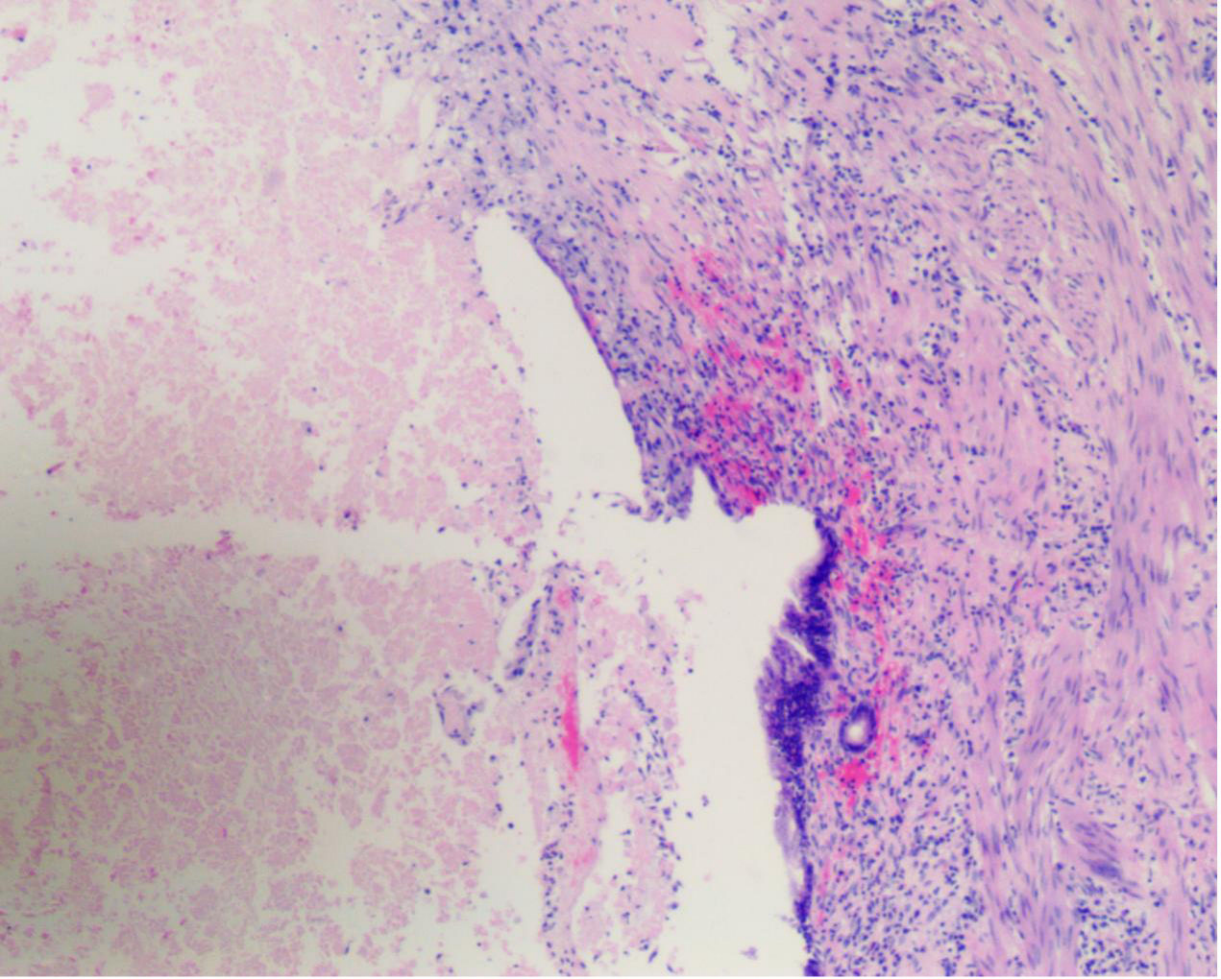
Posttreatment surgical specimen. H&E-stained sections from surgical specimens obtained after completion of the multimodal therapy regimen (radiotherapy, lenvatinib, and PD-1 inhibitor) revealed a complete pathological response (pCR). There are no viable tumor cells present, and the area previously occupied by the tumor is replaced by fibrotic tissue and chronic inflammatory infiltrate (arrows indicate areas of fibrosis and inflammation), indicative of a complete response to the treatment. Magnification: ×20.

On September 26, 2023, the patient underwent surgical resection, which included resection of hepatic segments 4b+5, gallbladder, extrahepatic bile duct resection, and laparoscopic lysis of adhesions. The procedure was completed smoothly in 8 hours. Postoperative pathology revealed no viable tumor cells in the gallbladder, liver, or common bile duct, which was consistent with the posttreatment response; there was no evidence of intravascular tumor emboli or nerve invasion; the pericholecystic lymph nodes were 0/3, and the lymph nodes of Groups 8 and 12 were 0/5. According to the RECIST criteria, the patient achieved complete remission (**Figure 4**).

## Discussion

This case represents the initial application of combined radiotherapy with targeted and ICI approaches as a preoperative conversion treatment for gallbladder cancer followed by surgical resection after downstaging in the international medical literature. Following the integrated treatment protocol, the tumor exhibited a significant reduction in size and was successfully downstaged. Postoperative pathology indicated a complete pathological response (pCR) with R0 resection and no increase in postoperative complications. This instance demonstrates the feasibility of a preoperative conversion therapy regimen comprising radiotherapy combined with lenvatinib and a PD-1 inhibitor followed by surgical resection 6 to 8 weeks posttreatment as a viable strategy for the management of advanced gallbladder cancer.

Gallbladder cancer is notorious for its insidious onset, with the majority of patients being diagnosed at an advanced stage when the opportunity for curative resection is critical or lost (2). Surgical intervention represents the cornerstone of treatment for gallbladder cancer, and radical resection offers the sole chance for long-term survival, significantly prolonging the survival of patients who achieve R0 resection status postoperatively (3). Both domestic and international experts concur that preoperative conversion therapy is indicated for patients with locally advanced or recurrent gallbladder cancer. The National Comprehensive Cancer Network (NCCN) guidelines recommend considering preoperative conversion therapy for gallbladder cancer patients with T3/T4 staging or lymph node metastasis. A meta-analysis demonstrated that overall survival (OS) is significantly prolonged in patients who undergo radical R0 resection following conversion therapy (4). Conversion therapy for gallbladder cancer remains in the exploratory phase, lacking a standardized treatment protocol, with chemotherapy regimens primarily based on gemcitabine currently predominating.

### Chemotherapy

In the context of chemotherapy, a retrospective analysis of the American Cancer Database revealed that the utilization of neoadjuvant chemotherapy among gallbladder cancer patients is relatively low. However, for patients with lymph node-positive gallbladder cancer, neoadjuvant chemotherapy significantly extends survival compared with direct surgery or surgery combined with adjuvant chemotherapy (6). A phase III ABC-02 study (7) established a combination of gemcitabine and cisplatin (GC) as a first-line treatment for unresectable biliary tract cancers.

A retrospective study by Chaudhari et al., which featured a substantial sample size of 160 patients with advanced gallbladder cancer, reported that 93 (58%) patients were candidates for surgery following GC chemotherapy. Among these patients, 63 (39.4%) achieved R0 resection, and the median overall survival (OS) for patients with R0 resection was significantly longer than that for patients who did not undergo conversion surgery (49.0 months vs. 7.0 months) (8). With an increasing array of first-line chemotherapeutic options for advanced biliary tract cancer, other regimens have also demonstrated promising results in surgical conversion.

A multicenter phase II study reported by Chinese scholars reported an objective response rate (ORR) of 48% with the combination of gemcitabine and nab-paclitaxel for the treatment of advanced gallbladder cancer, with surgical conversion achieved in 30% of patients (9). Furthermore, a study reported by S. Gedela et al. in 2023 retrospectively analyzed 142 patients with locally advanced biliary tumors, 52 of whom had gallbladder cancer. The ORR of patients treated with the combination of GC and nab-paclitaxel reached 67.6%, with 17 (34%) gallbladder cancer patients receiving radical surgery (10).

Additionally, the three-drug regimen FOLFIRINOX (irinotecan, 5-fluorouracil, combined with oxaliplatin) may improve the ORR compared with two-drug regimens such as GC and GEMOX (gemcitabine combined with oxaliplatin). However, the impact of FOLFIRINOX on enhancing the benefits of surgical conversion and survival remains to be determined (11, 12).

### Targeted therapy

In the domain of targeted therapy for gallbladder cancer, large-scale randomized clinical trials are lacking. Prior reports on treatments targeting VEGFR and MET have not yielded satisfactory outcomes for patients with gallbladder cancer (13, 14). However, the complex signaling pathways involved in gallbladder cancer suggest the potential for other targeted therapeutic approaches.

One promising avenue in recent years is HER2-targeted therapy. The multicenter phase 2b single-arm HERIZON-BTC-01 study reported at the 2023 ASCO Annual Meeting enrolled 80 patients with advanced biliary tract cancer, nearly half of whom had gallbladder cancer. Treatment with the HER2 dual-antibody zanidatamab achieved an objective response rate (ORR) of 41.3%, a median progression-free survival (PFS) of 5.5 months, and tumor shrinkage in approximately 68.4% of patients (15).

### Immune checkpoint inhibitors

Phase III studies, such as TOPAZ-1, KEYNOTE-966, and IMbrave151, have revolutionized clinical practice for advanced biliary tract cancer, leading to improvements in overall survival (OS) (16). However, the impact on the objective response rate (ORR) has been modest; for example, TOPAZ-1 reported an increase in the ORR from 18.7% to 26.7%, whereas KEYNOTE-966 reported no change in the ORR (17). Notably, targeted combined ICI protocols for advanced biliary tract tumors have shown promising efficacy without the need for chemotherapy. A study by Chinese researchers reported an ORR and median OS of 80% and 22.5 months, respectively, when a first-line GEMOX regimen combined with toripalimab and lenvatinib was used for advanced intrahepatic cholangiocarcinoma (18). In another retrospective study of targeted combination ICIs for advanced gallbladder cancer, the combination of toripalimab and lenvatinib achieved an ORR and disease control rate (DCR) of 32.3% and 83.9%, respectively, with a median progression-free survival (PFS) and median OS of 5.0 months and 11.3 months, respectively, and surgical conversion was achieved in 3 patients (9.6%) (19). A 2023 retrospective study reported an ORR of 46.2% for the combination of camrelizumab and lenvatinib in 52 patients with advanced gallbladder cancer, with a median PFS and median OS of 7.0 months and 12.0 months, respectively, suggesting an alternative for patients who cannot tolerate chemotherapy (20).

### Radiotherapy

Reports on the use of radiotherapy are rare. A preliminary study by Japanese researchers revealed that the combination of arterial infusion chemotherapy and external beam radiotherapy (IAC + RT) for biliary tract cancer resulted in an imaging response rate of 57.1%, a clinical response rate of 71.4%, and a disease control rate of 100% (21), indicating promising potential for translational therapy. In 2016, Agrawal et al. (22) published a retrospective study on chemoradiotherapy for unresectable gallbladder cancer in which 40 patients underwent translational therapy and 6 achieved R0 resection. In the same year, Engineer et al. (23) retrospectively analyzed the outcomes of gemcitabine combined with radiotherapy for locally advanced gallbladder cancer, with 14 out of 28 patients achieving R0 resection.

### Combined treatment programs

In recent years, interest in the combination of radiotherapy with targeted therapy and ICIs has increased, as evidenced by a growing body of preclinical and clinical research. A substantial amount of this research indicates that radiotherapy can induce the release of antigens and improve the tumor microenvironment, leading to the upregulation of the immune checkpoints PD-1 and CTLA-4 on immune cells. Additionally, radiotherapy enhances the expression of PD-L1 on tumor cells and increases VEGF expression. ICIs complement these effects by increasing T-cell activity and alleviating immune suppression. Targeted therapy contributes to vascular normalization, increases CD8+ T-cell infiltration, reduces tumor-associated macrophage (TAM) and regulatory T-cell (Treg) infiltration, and elevates PD-1 expression. The synergistic application of these three modalities has yielded promising therapeutic outcomes (24–28).

A phase I clinical trial combining radiotherapy with cetuximab and ipilimumab for locally advanced squamous cell carcinoma of the head and neck conducted by Ferris et al. (29) reported 3-year disease-free survival (DFS) and overall survival (OS) rates of 72% each, with definitive efficacy and no dose-limiting toxicities. In another study by Wang et al. (30) involving hepatocellular carcinoma patients with portal vein tumor thrombus treated with radiotherapy in combination with atezolizumab and bevacizumab (T+A), a cohort of 30 patients demonstrated an objective response rate (ORR) of 76.6% and a disease control rate (DCR) of 96.7%. The median overall survival (OS) was 9.8 months, and the median progression-free survival (PFS) was 8.0 months, with the median time to disease progression not yet reached. These studies collectively suggest that the combination of radiotherapy with targeted and immunotherapies offers encouraging therapeutic efficacy and acceptable safety profiles, presenting a viable treatment option for advanced gallbladder cancer.

This case employed a novel preoperative conversion strategy for critically resectable gallbladder cancer, combining radiotherapy with targeted and immunotherapies, which, to our knowledge, is the first of its kind in the international medical literature. Posttreatment, the tumor demonstrated a significant reduction in size and successful downstaging, and complete pathological remission (pCR) was achieved according to postoperative pathology. A case report highlights a promising treatment approach for advanced gallbladder cancer using a combination of chemotherapy and immunotherapy. The patient achieved a complete pathological response, suggesting that such combined strategies may offer effective alternatives to conventional therapies in select cases. The study supports the evolving role of chemo-immunotherapy as a potentially transformative option for improving outcomes in aggressive gallbladder cancer, though further research and clinical trials are needed to validate these findings (31). Another case report presents a rare case of complete response in gallbladder cancer using a combination of gemcitabine and cisplatin chemotherapy with durvalumab. This case suggests that adding immunotherapy to standard chemotherapy may offer promising outcomes for advanced gallbladder cancer (32). Notably, there was no increase in postoperative complications, and any adverse reactions encountered during the treatment were tolerable. The successful conversion in this patient suggests that the trimodal approach of radiotherapy combined with targeted and immunotherapies may offer a promising therapeutic option. We anticipate that future prospective studies will further validate this potential.

## Conclusions

Radiotherapy combined with lenvatinib and PD-L1 inhibitors can successfully achieve R0 resection of critically resectable gallbladder cancer, with good results and tolerable toxicities.

## Data Availability

The raw data supporting the conclusions of this article will be made available by the authors, without undue reservation.
